# One-pot nanoflower-based sensitive colorimetric biosensor for multihost detection of zoonotic clonorchiasis

**DOI:** 10.1371/journal.pntd.0014197

**Published:** 2026-04-13

**Authors:** Xiaoxiao Ma, Qizhi Liu, Qiaocheng Chang, Qingbo Lv, Lei Zhang, Shumin Sun, Chunren Wang, Mingyuan Liu, Xiaolei Liu, Chen Li

**Affiliations:** 1 State Key Laboratory for Diagnosis and Treatment of Severe Zoonotic Infectious Diseases, Key Laboratory for Zoonosis Research of the Ministry of Education, Institute of Zoonosis, and College of Veterinary Medicine, Jilin University, Changchun, China; 2 College of Animal Science and Technology, Yunnan Agricultural University, Kunming, China; 3 College of Life Sciences, Hunan Normal University, Changsha, Hunan, China; 4 School of Public Health, Shantou University, Shantou, Guangdong, China; 5 College of Animal Science and Veterinary Medicine, Heilongjiang Bayi Agricultural University, Daqing, China; 6 Jiangsu Co-innovation Center for Prevention and Control of Important Animal Infectious Diseases and Zoonoses, Yangzhou University, Yangzhou, Jiangsu, China; University of Oxford, UNITED KINGDOM OF GREAT BRITAIN AND NORTHERN IRELAND

## Abstract

*Clonorchis sinensis*, a significant but frequently overlooked foodborne zoonotic helminth, is responsible for infecting approximately 15 million individuals globally, poses an infection risk to nearly 200 million people, and represents a threat to public health while imposing considerable economic burdens. The broad diversity and widespread distribution of definitive hosts significantly contribute to the sustained prevalence of clonorchiasis, while the diagnostic challenges are further exacerbated by the intricate variability in host species and their geographical dispersion, alongside the absence of accurate multihost detection techniques, ultimately driving increasing disease incidence. In this study, we fabricated a one-pot nanoflower-based sensitive competitive ELISA (nano-cELISA) for the multihost detection of clonorchiasis. The functional organic-inorganic hybrid nanoflowers retain the ability to specifically bind to the *C. sinensis* tandem repeat sequence 1 (CSTR1) antigen and leverage the catalytic function of horseradish peroxidase, thereby facilitating signal amplification while ensuring specificity. The detection performance of the colorimetric biosensor was systematically assessed via multihost serum samples (humans, dogs and mice). Seroconversion in artificially infected mice and dogs occurred at 21 days post-infection (dpi), indicating high specificity without cross-reactivity with sera from other parasitic infections. The nano-cELISA demonstrated a sensitivity of 100% and a specificity of 91.67% in clinical serum samples from dogs and 95.49% sensitivity and 96.72% specificity in human samples. The developed nano-cELISA method has straightforward preparation and operational simplicity, representing a useful tool for epidemic containment and offering a robust technical strategy for the prevention and control of *C. sinensis*-infection.

## Author summary

This study presents a nanoflower-enhanced competitive ELISA developed for multi-host serodiagnosis of clonorchiasis, a neglected yet substantial food-borne parasitic disease affecting millions worldwide. The assay integrates organic-inorganic hybrid nanoflowers to improve detection sensitivity through signal amplification while preserving high specificity toward the CSTR1 antigen. Evaluated across human, canine, and mice sera, the platform demonstrated early seroconversion at 21 dpi in experimental models, with no cross-reactivity to other parasites. It achieved high sensitivity (100% in dogs, 95.49% in humans) and specificity (91.67% in dogs, 96.72%in humans). With simple preparation and reliable performance, this nano-cELISA provides a practical tool for large-scale screening and control of clonorchiasis.

## Introduction

Foodborne trematodes pose a significant global health threat by infecting animals and humans through the ingestion of food products, notably raw fish and crustaceans [1–2]. *Clonorchis sinensis,* the primary etiological agent of *C. sinensis*-infection, infects approximately 15 million individuals globally and poses an infection risk to nearly 200 million people, predominantly in Asian regions, with China, Vietnam, Korea, and Japan being the most significantly affected. *C. sinensis* was designated a Group I carcinogen by the International Agency for Research on Cancer (IARC), underscoring its critical importance as a major public health concern [3]. *C. sinensis* infection is associated with a range of hepatobiliary disorders, including cholangitis, periductal fibrosis, and the development of liver cirrhosis [4]. Comprehensive studies have established a clear link between *C. sinensis* infection and the onset of cholangiocarcinoma, emphasizing the gravity of its health impacts and the pressing need for effective detection methods and preventive interventions [5].

The intricate life cycle, characterized by a sophisticated three-host system encompassing a primary intermediate host (snails), a secondary intermediate host (freshwater fishes), and a definitive host, including humans and various animal reservoirs such as cats, pigs, dogs, minks, badgers, and mice, significantly increases the challenges associated with the prevention and control of *C. sinensis*-infection [6]. Research on the detection of *C. sinensis*-infection has focused predominantly on fish and humans; however, the persistence of numerous preserved host species, coupled with inadequate detection measures, may sustain the transmission of the pathogen [7–8]. Completely eliminating *C. sinensis*-infection through detection and prevention strategies focused solely on a single host (such as humans or dogs) remains challenging, particularly in certain provincial administrative regions [9]. Therefore, the development of a multihost detection strategy for the epidemiological surveillance of clonorchiasis, encompassing infection rates across diverse food chain hosts, is highly important for achieving a comprehensive and multifaceted approach to disease prevention and control.

Serological detection methods, particularly enzyme-linked immunosorbent assays (ELISA), can be utilized for the detection of multiple hosts, especially for the rapid diagnosis of diseases at various scales [10]. Traditional ELISA methods face obstacles, particularly in terms of limited sensitivity and instability, which are primarily attributed to the intrinsic properties of bioenzymes, suboptimal enzyme‒antibody conjugation, and the inhibitory effects of immobilization on immunoreactions [11]. Taking *C. sinensis* as an example, although excretory-secretory products (ESPs) and worm crude antigen (CA) are the most widely employed antigens for detection via ELISA, considerable room remains for enhancement in terms of specificity and sensitivity [12]. Antigens featuring tandem repeat sequences (TRSs) frequently act as targets for B-cell-mediated responses and have demonstrated significant utility in the serological diagnosis of various parasitic infections, such as those caused by *F*. *hepatica*, *S*. *mansoni, C. sinensis*, and *Trypanosoma cruzi* [13]. With respect to *C. sinensis*, proteins such as CsGRP [14], CsPRA [15], Cs1 [13] and Cs31 [16] have been documented to possess TRs and exhibit effective detection capabilities. Notably, in contrast to previous antigenicity studies focused solely on a single host, CSTR1 has demonstrated its proficiency in detecting infections across multiple hosts, thereby substantially increasing its potential for broader application.

Conventional ELISAs rely on natural enzymes such as horseradish peroxidase (HRP), which are susceptible to inactivation under suboptimal conditions and involve complex, time-consuming preparation procedures. These limitations can restrict their reproducibility and applicability in resource-limited or multi-host surveillance settings. Fortunately, extensive research has been conducted across various domains, ranging from the streptavidin‒biotin system to nanomaterials, aimed at enhancing the performance of ELISA [17]. The sensitivity and stability of ELISA have notably improved, particularly in the context of nanomaterial-based ELISA (nano-ELISA) [18]. Nanostructured materials, which are increasingly employed as carriers for enzyme immobilization, are favored because of their distinctive and adjustable characteristics [19]. Within this category, a newly emerged type of protein-inorganic hybrid nanostructure has garnered considerable attention in recent years owing to its mild synthesis conditions (room temperature, one-pot method), elevated efficiency, and capacity to stabilize enzymes, representing an increasingly common strategy for enzyme immobilization [20–22]. Protein-inorganic hybrid nanoflowers have undeniably laid a robust groundwork for the advancement of nano-ELISA, with a diverse range of detection methods employed to identify various pathogens, including bacteria, viruses, and parasitic organisms [23–26].

We hypothesized that a protein-inorganic hybrid nanoflower, incorporating both a specific monoclonal antibody and HRP, could serve as a stable and highly catalytic detection reagent. Building on this hypothesis, the primary objective of this study was to develop and preliminarily evaluate a nanoflower-based competitive ELISA (nano-cELISA) for the detection of *C. sinensis*-infection across multiple host species. The performance of this devised nano-cELISA system, including its preparation and operational characteristics, was assessed to explore its potential applicability in supporting clonorchiasis surveillance.

## Materials and methods

### Ethics statement

This study was approved by the Ethical Committee of the First Hospital of Jilin University, (ethical clearance number # 2021–703). Following the acquisition of written informed consent, serum samples were collected from all adult participants and conducted according to the guidelines of the Declaration of Helsinki.

All animal experiments conducted in this study were authorized by the Ethics Committee of Jilin University (Protocol # 20170318), which is affiliated with the Provincial Animal Health Authority in China.

### Parasites and sera

The metacercariae of *C. sinensis* were isolated from the muscle tissues of naturally infected *Pseudorasbora parva* collected from the epidemic area of Changchun city, Jilin Province, by artificial digestion according to our previous method [27]. Serum samples positive for *C. sinensis*-infection were generated through artificial infection of experimental animals. Specifically, three beagles (female, 8 month), Six New Zealand White rabbits (female, 2 month), and 12 BALB/c mice (female, 6 weeks), sourced from the Jilin University Experimental Animal Facility, were orally inoculated with 500, 200, and 20 viable metacercariae, respectively. Blood samples for serum collection were taken one week before inoculation and then weekly post-inoculation. Serum samples collected from dogs infected with other prevalent parasitic organisms (n = 4), namely, *Metorchis orientalis*, *Echinochasmus japonicus*, *Toxocara canis* and *Toxoplasma gondii,* were archived at our institution.

All animal experiments conducted in this study were authorized by the Ethics Committee of Jilin University (Protocol # 20170318), which is affiliated with the Provincial Animal Health Authority in China.

A total of 133 clinical adult human serum samples were utilized. Specifically, 59 serum samples were obtained from patients diagnosed with *C. sinensis*-infection and confirmed to be egg positive via the Kato-Katz (KK) technique, whereas 74 serum samples were sourced from healthy individuals who were free of parasitic infections. Serum samples from nine patients infected with various parasites, including *Echinococcus granulosus*, *Toxoplasma gondii*, *Paragonimus westermani*, *Schistosoma japonicum*, *Taenia solium*, *Trichinella spiralis*, *Trichuris trichura*, *Ascaris suum* and *Ascaris lumbricoides*, were acquired from the Chinese Center for Disease Control and subsequently archived at our institution.

### Reagents and antibodies

The monoclonal antibody (McAb) targeting CSTR1, which was generated in mice and subsequently purified via a staphylococcal protein A column (custom-produced by Jiangsu Nanjing Yuantai Biotechnology Co., Ltd.), was maintained in our laboratory. Tetramethyl benzidine (TMB), horseradish peroxidase (HRP) and CuSO_4_ ⋅ 5H_2_O were obtained from Solarbio (Beijing, China). HRP-conjugated antibodies against rabbit IgG, mouse IgG, dog IgG, and human IgG were obtained from Beijing Biolab Technology (Beijing, China).

### Expression of CSTR1 antigen and western boltting

The CSTR1 antigen comprises a repetitive sequence defined by uniform repeating units. Each of these units, when translated, corresponds to an amino acid sequence of (GGKAPPPESA) [28]. A sequence comprising 10 repetitive units was synthesized and then fused to the pET-41a vector, which included a GST tag, to construct a recombinant plasmid, and the sequence was synthesized by the Sangon Biotech (Shanghai) Co., Ltd. Following induction and expression, purification was conducted utilizing a GST-Tag protein purification kit with magnetic agarose beads (Beyotime Biotechnology, Shanghai), adhering strictly to the manufacturer’s protocol. Western blotting was subsequently utilized to validate the detection efficacy of CSTR1 against both positive and negative *C*. *sinensis* serum samples from dogs, rabbits, and humans.

### Working principle of the nano-cELISA protocol

**Fig 1:**
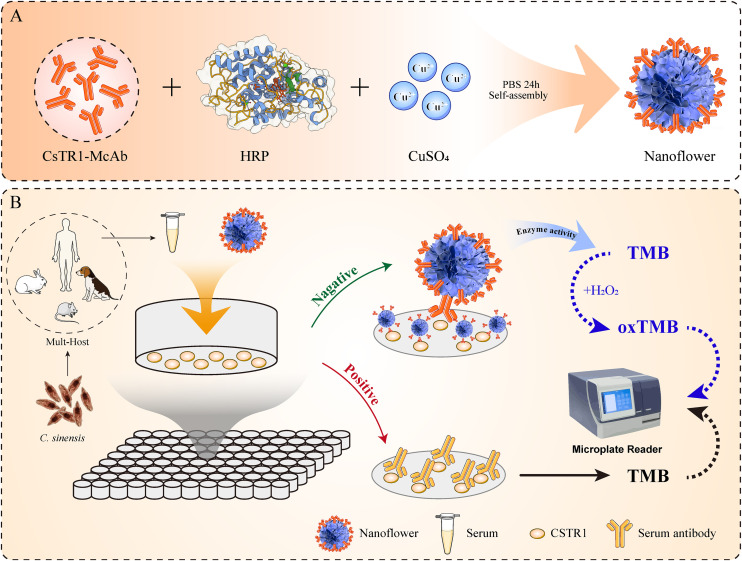
The principle of the nanoflower based-competitive ELISA immunoassay. **(A)** Preparation of functional nanoflowers. **(B)** The procedure for detecting using the nano-cELISA method.

The fundamental operating mechanism of the nano-cELISA experiment involves the following key components: initially, McAb, HRP, and Cu^2+^ spontaneously self-assemble in a phosphate-buffered saline (PBS) solution to form functional nanoflowers (Fig 1A). Then, the ELISA plate was coated with the CSTR1 antigen. Following sealing, both the serum sample under investigation and the functional nanoflower were concurrently introduced into the reaction wells. In negative samples, the nanoflower fully engages with the antigen, causing a detectable color change of the TMB from transparent to deep blue, as measured by a microplate reader. In contrast, in positive samples, the CSTR1-antibody in serum competes with the nanoflower for antigen binding sites, resulting in decreased substrate catalysis and a lighter coloration. Finally, the determination for each sample was represented as the competitive inhibition (PI) ratio [29], defined mathematically as follows: PI = (1-OD_sample_/OD_nanoflower_) × 100%, where OD_sample_ and OD_nanoflower_ denote the optical densities of the sample and the nanoflower control, respectively (Fig 1B).

### Preparation, characterization and verification of the McAb-HRP@Cu-HNFs

The nanoflowers were prepared via the following techniques: initially, McAbs and HRP were diluted to a concentration of 1 mg/mL, and 300 μL of each diluted solution was added to 500 mL of PBS (0.1 M) [30]. Subsequently, 100 μL of CuSO_4_ (100 mM) solution was added, and the resulting mixture was allowed to incubate at room temperature for 24 h [31]. Nanoflowers progressively develop into floral-like configurations, utilizing metal ions as nucleation points for their growth. As the quantity of nanoflowers increased, the entire solution was centrifuged at 12,000 rpm for 3 min to facilitate the harvesting of the crystalline precipitate. This precipitate was then rinsed three times with ddH_2_O and subsequently dried at 30°C to yield a solid crystalline powder, termed McAb-HRP@Cu-HNFs [32–33]. An identical experimental protocol was utilized to synthesize the McAb@Cu-HNFs and HRP@Cu-HNFs. Each of the three nanoflower preparations was diluted to a concentration of 1 mg/mL. Subsequently, 30 μL aliquots of each solution were deposited onto pristine silicon wafers and permitted to dry spontaneously. After surface gold coating, the wafers were placed under a benchtop scanning electron microscope to observe the microscopic morphologies.

To achieve optimal performance of the nanoflowers through an ideal blend of constituents, the concentration of Cu^2+^, the proportion of McAb to HRP, and the temporal evolution of natural complexation within the nanoflowers were meticulously optimized. These parameters were then integrated into the detection system for *C. sinensis*-infection. In addition, the enzymatic activity of the McAb-HRP@Cu-HNFs was assessed by examining both the binding capability of the monoclonal antibody and the catalytic efficiency of the enzyme-active components toward the substrate within the HNFs.

### Optimization of nano-cELISA

Several factors have been recognized as impacting the efficacy of nano-cELISA, thus necessitating thorough optimization of the reaction system [34–35]. In this study, the optimal concentration of antigen and the appropriate antibody dilution were determined using a checkerboard method. The dilution ratio of the nanoflowers and their detection ratio in serum were subsequently further optimized. Additionally, other parameters, such as the antigen amount, coating solution, and duration, blocking solution and duration, competitive incubation period and substrate reaction time, were also adjusted to enhance the overall performance. The optimal conditions were determined on the basis of the highest PI value achieved.

### Evaluation of the nano-cELISA performance

To test the feasibility and practicality of the established method, serum samples and corresponding fecal samples were tested separately. Human, dog and mouse sera were evaluated using optimized nano-cELISA, and the fecal sample corresponding to each serum sample was simultaneously measured via the KK method [36]. The cutoff value for each host species in the assay was established using a panel of negative control sera. Considering the normal distribution evident in the frequency histograms of the negative serum PI ratios for each species, the cutoff value for each species was calculated as the mean PI value of the negative controls plus two standard deviations.

### Assay on specificity of nano-cELISA

The antibody kinetics of *C. sinensis* infection were assessed in dog and mouse models across various infection periods, while the cross-reactivity of the nano-cELISA was evaluated utilizing sera from individuals infected with other parasites. Seroconversion was delineated as the point in time when the *C. sinensis* antibody concentration surpassed the cutoff PI ratio. The diagnostic utility of the nano-cELISA was evaluated through the use of 133 human and 53 dog serum clinical samples randomly selected from Fuyu County in Jilin Province, China, an endemic area for clonorchiasis.

### Statistical analysis

All data were statistically analyzed using GraphPad Prism 9 (GraphPad Software, USA), and different groups were analyzed using one-way analysis of variance (ANOVA). All experiments were repeated at least three times, and data are expressed as the mean ± standard error. Differences were considered statistically signiﬁcant when the *P-*value was < 0.05, ****P < 0.0001, ***P < 0.001, **P < 0.01, *P < 0.05.

## Results and discussion

### Antigen validation

In our prior research, the CSTR1 antigen exhibited promising potential for detecting *C. sinensis*-infection [29]. This antigen was successfully expressed using a prokaryotic system, yielding a soluble protein of approximately 40 kDa in size, which included a GST tag (S1A Fig). GST monoclonal antibodies were utilized to detect both the control empty vector pET41a and the purified CSTR1 antigen, with distinct recognition of the latter (S1B Fig). Western blotting analysis of sera from multiple hosts revealed that the CSTR1 antigen could be recognized by sera from humans, dogs, and mice infected with clonorchiasis (S1C Fig).

### Characterization of nanoflower microstructure

HRP, a prominent and extensively utilized enzyme, has garnered particular attention owing to its versatile catalytic capabilities across diverse conditions [37]. Therefore, we utilized the enhanced detection ability of HRP in combination with the nanoflower component in the experiment to achieve satisfactory results. Proteins and HRP interact with Cu² ⁺ , resulting in the formation of crystalline nanoparticles, and then, the precipitation process begins with the emergence of primary calcium phosphate crystals [38]. During this stage, protein molecules serve as nucleation centers for these nascent crystals, facilitating the assembly of larger clusters that encompass both protein molecules and these primary crystals [39]. As illustrated in S2 Fig and 3D nanoflowers, namely, HRP@Cu-HNFs, McAb@Cu-HNFs, and McAb-HRP@Cu-HNFs, were synthesized through a mild, self-assembly based biomimetic approach. Scanning electron microscopy (SEM) of these products revealed flower morphologies: HRP-HNFs displayed a looser structure with a larger specific surface area; McAb@Cu-HNFs exhibited the most compact morphology, resembling a rose; and McAb-HRP@Cu-HNFs featured a ‘chrysanthemum-like’ structure and had the largest surface area among the three. In terms of detection capabilities, HRP-HNFs can catalyze substrate color development. When incorporated into a cELISA system, HRP does not compete for binding with antibodies in positive control serum, thereby exerting no influence on the detection results (S2A Fig). McAb@Cu-HNFs exhibit specific antibody recognition ability and can achieve specific competitive binding to CRTR1 antibodies in serum, albeit with a relatively low PI ratio (S2B Fig). McAb-HRP@Cu-HNFs, with their dual functionality, can enhance the detection effectiveness. Compared with the McAb@Cu-HNFs, the McAb-HRP@Cu-HNFs also enable specific competitive binding to CRTR1 antibodies in serum, and they exhibit a significantly improved PI ratio, outperforming the McAb@Cu-HNFs alone (S2C Fig).

### Nanoflower synthesis and characterization

**Fig 2:**
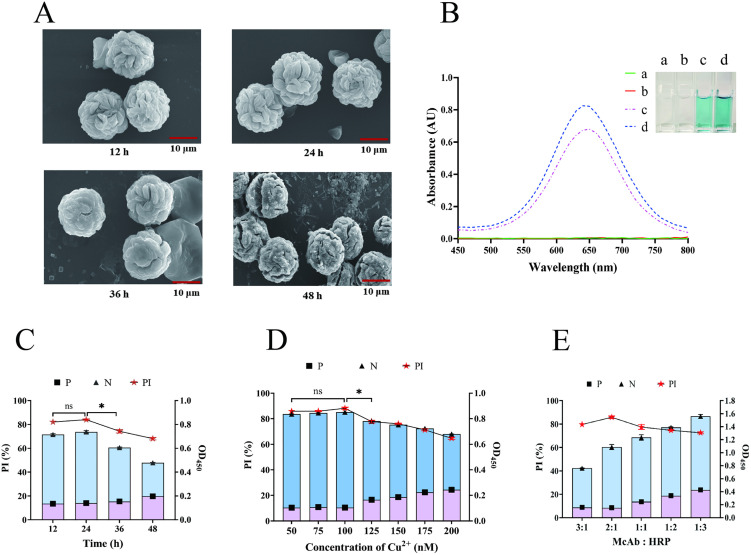
Optimization of the generating conditions for McAb-HRP-HNFs. **(A)** The morphology of McAb-HRP@Cu-HNFs observed under SEM at 12-48h. **(B)** Verification of catalytic ability of nanoflowers. a, blank control of reaction substrate (TMB + H_2_O_2_). b, McAb and reaction substrates. c, HRP and reaction substrates. d, functional nanoflowers and reaction substrates. **(C)** The detection effect of the nanoflowers at different growth stages. **(D)** Screening the optimal Cu^2+^ concentration. **(E)** Screening of the proportion of McAb and HRP (*: *P <* 0.05, ns: no significant difference).

Nanoflowers play crucial roles in the detection system; thus, the conditions for their generation were optimized [40–41]. The microscopic morphologies and detection efficacies of the nanoflowers were examined via SEM at 12, 24, 36, and 48 hours. From 12 to 24 h, the overall structure of the nanoflowers remained intact and loose, indicating a large specific surface area. Between 36 and 48 h, the morphology of the nanoflowers gradually became compact, with some binding sites becoming closed. The detection efficiency at 12–24 hours was significantly greater than that at 36–48 hours (Fig 2A). Compared with McAbs, both HRP and functional nanoflowers can catalyze substrate discoloration (TMB + H_2_O_2_) (Fig 2B). The amount of nanoflowers generated at 24 h was notably greater than that generated at 12 h. Considering both the maximum utilization rate and detection efficacy, McAb-HRP@Cu-HNFs grown for 24 h were ultimately selected (Fig 2C).

An appropriate concentration of Cu^2+^ can achieve maximum utilization of the raw materials and avoid interference from excess ions [42]. Seven concentrations of Cu^2+^ were established, ranging from 50 nM to 200 nM. The detection outcomes at concentrations between 50 nM and 100 nM were notably superior to those at concentrations between 125 nM and 200 nM, with no statistically significant difference observed within the 50 nM to 100 nM range. However, the nanoflower yield was greater at 100 nM. Consequently, the optimal concentration of Cu^2+^ was identified as 100 nM (Fig 2D). In the functionalized hybrid nanoflower system, McAb can specifically recognize antigens, while HRP can enhance catalytic reactions. An appropriate ratio of McAb to HRP can enhance their synergistic effect. Five ratios of McAb:HRP were used: 3:1, 2:1, 1:1, 1:2, and 1:3. The results revealed that the detection efficacy was optimal when the addition ratio of McAb:HRP was 2:1 (Fig 2E).

### Catalytic activity of the nanoflowers

A relatively ideal nanoflower exhibits catalytic abilities that rival those of natural enzymes while also possessing improved stability [43]. To assess the peroxidase-like activity of the HNFs following successful synthesis, the system was utilized with substrates (TMB and H_2_O_2_) to initiate redox reactions, thereby verifying its efficacy in detecting sera from various hosts. Initially, the optimal antigen coating concentration and antibody dilution ratio were determined via the checkerboard method [44]. When the CSTR1 antigen was coated at 7.5 μg/mL and the ratio of rabbit serum to McAb-HRP@Cu-HNFs was 1:2, optimal detection performance was achieved (S1 Table). Then, the sera of rabbits, mice, dogs, and humans were diluted with PBS at ratios of 4:1, 3:1, 2:1, 1:1, 1:2, 1:3, and 1:4, respectively. The optimal ratio was found to be 1:2 (S3A–S3D Figs). The optimal storage pH (in the pH range of 3–12) was also determined. McAb-HRP@Cu-HNFs exhibited low activity (<20%) at pH values of 3–4. The highest activity (>95%) was observed at a pH of 5. When the pH reached 8, the activity decreased to approximately 55%. In addition, over 75% of the storage stability of the McAb-HRP@Cu-HNFs was retained within 30 days at room temperature, and over 90% of its activity was maintained when the material was stored at 4°C (S3F Fig). These results indicate that the synergistic interactions between proteins and Cu^2+^ within the hybrid three-dimensional nanoflowers could increase the stability and shelf-life of the detection system, which aligns with prior literature.

### Optimization of the detection conditions for nano-cELISA

**Fig 3:**
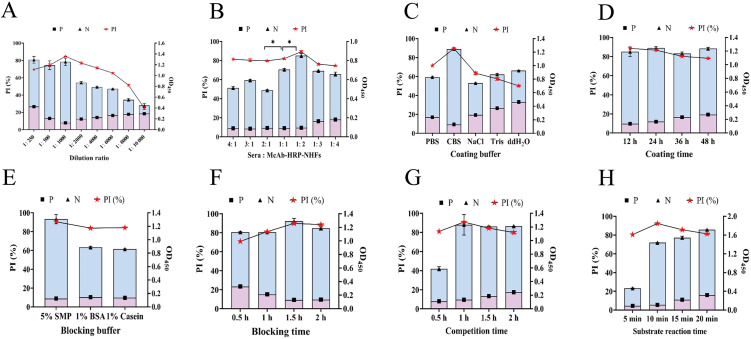
Optimization of the nano-cELISA reaction system. **(A)** Selection of optimal dilution ratio for McAb-HRP@Cu-HNFs. **(B)** Determination of the usage ratio between test serum and nanoflowers. **(C, D)** Optimization of coating buffer and coating time. **(E, F)** Optimization of blocking buffer and blocking time. **(G)** Optimization of the competition time. **(H)** Optimization of the substrate reaction conditions.

To establish a sensitive and robust nano-cELISA methodology, various crucial parameters, including the nanoflower dilution factor, coating conditions, blocking protocols, competitive incubation period, and substrate reaction duration, were meticulously optimized [13,20,26]. The optical PI ratios of the control group (rabbit serum negative for *C. sinensis* infection) and experimental group (rabbit serum positive for *C. sinensis* infection, 35 dpi) were measured to evaluate the impact of these factors. The optimal concentration of nanoflowers should yield an optimal signal-to-background ratio [45]. Consequently, a 1000-fold dilution was chosen as the optimal concentration, achieving a saturated signal-to-noise ratio (Fig 3A). At a detection ratio of serum to nanoflowers of 1:2, the PI value reached approximately 90%, indicating a significant deviation from neighboring concentrations (Fig 3B). Compared with other coating solutions, including PBS, NaCl, Tris, and ddH_2_O, CBS demonstrated optimal performance after 12 h of coating (Fig 3C and 3D), and the most suitable blocking conditions involved the use of 5% skim milk at 37°C for 1 h (Fig 3E and 3F). The results of the competitive incubation experiments revealed false positives in the control group when the incubation period for the nanoflowers exceeded 0.5 h because of nonspecific binding of the HNFs as a consequence of an extended incubation time, and the optimal effect was achieved after 1 h of incubation (Fig 3G). Finally, the optimized substrate reaction time was determined to be 10 min, with a PI value exceeding 95% (Fig 3H).

### Proof-of-Principle

To enable the detection of *C. sinensis*-infection across multiple hosts, a nano-cELISA detection system was established. The final operational procedure and interpretation criteria are as follows: CSTR1 was diluted with CBS to 7.5 μg/mL, and 100 μL per well was added for coating under 4 °C for 12 h, followed by washing three times (3 min each) with PBST under agitation. Then 200 μL of 5% skim milk (dissolved in PBST) was added per well for blocking, incubated at 37 °C for 1.5 h, and washed three times (3 min each) with PBST. McAb-HRP@Cu-HNFs were diluted 1:1000 with PBS; the test serum was mixed with McAb-HRP@Cu-HNFs at a ratio of 1:2, and 100 μL of the mixture was added per well. Competitive reaction was performed at 37 °C for 1 h, followed by washing three times with PBST and thorough drying of the plate. Subsequently, 100 μL of substrate solution (chromogenic solution containing 30% H₂O₂ and 50 mM TMB) was added per well, followed by incubation at 37 °C for 10 min for full color development. The reaction was terminated by adding 2 M sulfuric acid. The absorbance (OD₄₅₀) of each test sample and the McAb solution control were measured using a microplate reader, and the percentage inhibition (PI) value was calculated for each sample.

### Clinical validation

#### Preliminary validation of the nano-cELISA in humans.

**Fig 4:**
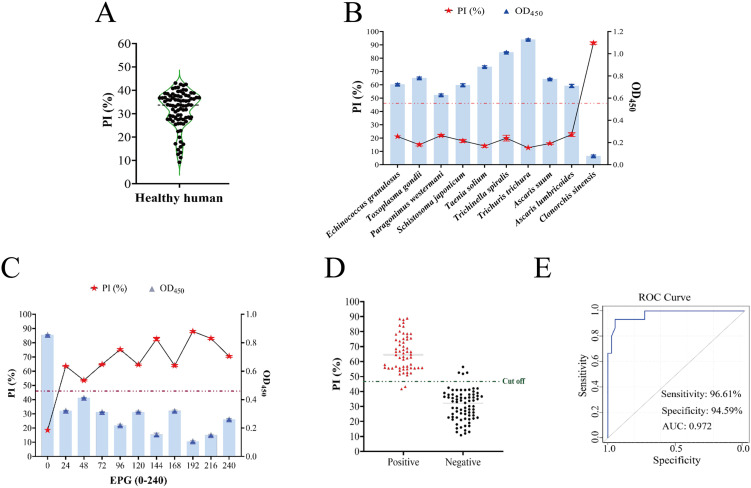
Preliminary validation of the nano-ELISA in humans. **(A)** Determination of the cut-off value for human sera. **(B)** Specificity tests. **(C)** Sensitivity test. **(D)** Detection of human clinical samples. **(E)** ROC curve analysis.

China boasts approximately 15 million instances of clonorchiasis; however, mild infections frequently remain asymptomatic, rendering this disease underrecognized [46]. To validate the effectiveness of nano-cELISA in humans, 88 serum samples from healthy individuals were evaluated. The calculated mean PI value was 31.8%, with a standard deviation of 7.87, and the predetermined cutoff value was set at 47.54% (Fig 4A). The performance of the method was subsequently systematically evaluated through specificity and sensitivity assessments as well as clinical sample testing. Specificity assessments were conducted using human serum samples containing sera from nine different parasite species. The findings revealed that the method demonstrated exclusive specificity for *C. sinensis*, without any cross-reactivity with sera from other parasitic infections, confirming its high specificity (Fig 4B). In sensitivity experiments, serum samples were categorized into different infection levels on the basis of the intensity of infection detected by the KK method in fecal samples (EPG: 0–240). The results showed that the method was capable of identifying human serum samples from individuals with varying intensities of infection, including those with low-intensity infections (EPG ≥ 24) (Fig 4C). A total of 133 human serum samples were assessed utilizing the enhanced nano-cELISA, resulting in the identification of 60 positive and 73 negative samples. Compared with the KK method, nano-cELISA displayed a sensitivity of 96.72% and specificity of 95.49% (Fig 4D and S2 Table) and demonstrated comparable performance to conventional ELISA techniques (S3 Table) [12,45,47–50].

#### Analytical performance of the nano-cELISA in dog sera.

**Fig 5:**
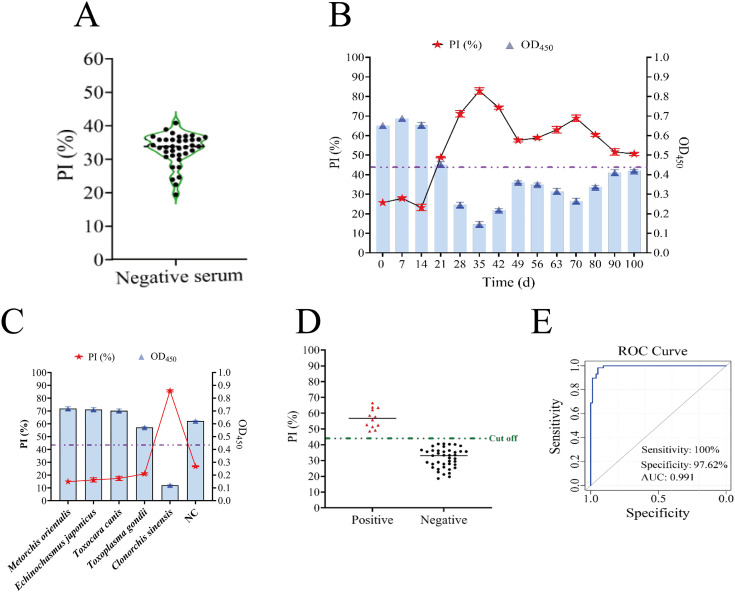
Analytical performance of the nano-cELISA in dog sera. **(A)** Determination of the cut-off value for dog sera. **(B)** Evaluation of seroconversion responses in dog after experimental infection with *C. sinensis*. **(C)** Specificity tests. **(D)** Detection of dog clinical samples. **(E)** ROC curve analysis.

As significant companion animals, dogs are closely intertwined with human living patterns, and the incidence of infection has remained fairly constant, with rates fluctuating from 22.6% before 1990 to 17.3% after 2010 [8]. Therefore, intensified surveillance of dogs, coupled with immediate deworming management, is highly important for prevention and control efforts. Under the aforementioned optimized conditions, dog serum samples were quantitatively assessed using the nano-cELISA system. A total of fifty-five negative serum samples were used to determine the mean PI value, which was 32.91%, accompanied by a standard deviation of 5.11, with a 43.13% cutoff value (Fig 5A). By testing serum samples collected from dogs artificially infected with *C. sinensis* at various time points post infection, an antibody titer curve was plotted, and antibody positivity was first detected at 21 dpi (Fig 5B). Additionally, when positive serum samples from dogs infected with different parasites were examined, nano-cELISA revealed no cross-reactivity with sera from other parasitic infections (Fig 5C). Finally, a comparison was conducted between the detection results of 53 canine clinical serum samples using the nano-cELISA and KK fecal examination. The nano-cELISA detected 12 positive and 41 negative samples, with a sensitivity of 91.67% and a specificity of 100% (Fig 5D and 5E and S2 Table).

#### Evaluation of the nano-cELISA using artificially infected mice serum.

**Fig 6:**
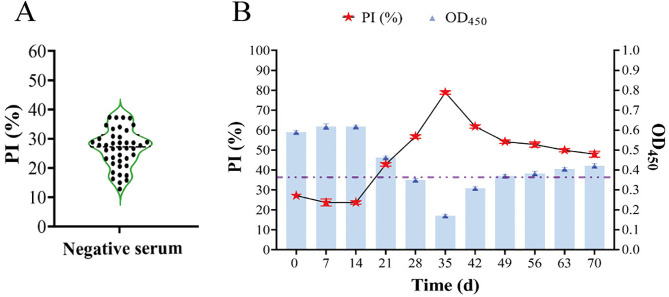
Evaluation of the nano-cELISA using artificially infected mice sera. **(A)** Determination of the cut-off value for mice sera. **(B)** Assessment of the seroconversion in mice after artificially infected with *C. sinensis*.

Mice are commonly used as experimental animal models for the study of the pathogenesis of *C*. *sinensis*, but they are also frequently disregarded as key reservoirs, with an infection rate of 3.6% [8]. In this study, we preliminarily evaluated the detection effect of nano-cELISA on mouse serum samples. To determine the cutoff value for positivity, 42 negative mouse serum samples were tested using the optimized nano-cELISA method. The PI values of the tested sera were calculated, yielding a mean of 25.63 with a standard deviation of 6.19. The cutoff value was 38.01% (Fig 6A). Antibody positivity was first detected at 21 dpi using this method by examining serum samples collected from mice artificially infected with *C. sinensis* at various time points post-infection (Fig 6B). The importance of mice as parasite reservoirs warrants attention, given their high probability of consuming raw fish, particularly discarded fish stranded due to drought-exposed riverbeds.

Presently, neither domestic nor international markets offer commercial products tailored for detecting *C. sinensis*-infection, significantly impeding efforts to prevent and manage this ailment [46]. When precision medical interventions are targeted among susceptible populations, monitoring the infection frequency of various definitive hosts within the food chain is equally imperative, achieving a multifaceted and comprehensive prevention and control objective. Accurate infection information is crucial for the epidemiological surveillance, prevention and control of this disease and represents a worthwhile area for future exploration. For the first time, this study established a multihost detection technique for *C. sinensis*-infection based on functional nanoflowers, which demonstrated favorable diagnostic performance and application potential.

While the developed nano-cELISA demonstrates passable performance, several limitations should be acknowledged. First, the clinical validation in this study, although showing high sensitivity and specificity, was conducted on a relatively limited number of samples from specific regions. Future studies involving larger-scale, multi-center, and geographically diverse cohorts are necessary to further confirm the robustness and generalizability of the assay across different epidemiological settings. Second, the current method primarily relies on serum samples, which requires a professional sampling process. Exploring its adaptability to more accessible sample types, such as whole blood or saliva, could significantly enhance its utility in field screening and resource-limited areas. Third, the nanoflower synthesis process, while straightforward, requires optimization for batch-to-batch consistency to ensure reproducible performance in large-scale production. Furthermore, although no cross-reactivity was observed with the tested parasitic sera, expanding the panel of cross-reactivity tests to include a wider range of regionally prevalent helminths would strengthen the specificity claim. Future work will also focus on simplifying the assay procedure further, potentially developing a lateral flow format based on the same nanoflower probe, to achieve a true point-of-care test. Addressing these aspects will be crucial for translating this laboratory-based technique into a practical, field-deployable tool for comprehensive clonorchiasis surveillance and control.

## Conclusion

This study has developed a nanoflower-based cELISA for the serological detection of *C. sinensis* infection across multiple host species. The one-pot self-assembly of functional organic-inorganic hybrid nanoflowers streamlines the preparation process, while effectively binding to the CSTR1 antigen and enabling catalytic signal amplification. The assay demonstrated favorable diagnostic performance in serum samples from humans, dogs, and mice, with no cross-reactivity observed against sera from other parasitic infections. Although these preliminary findings suggest potential utility for multi-host surveillance, several limitations warrant further investigation. Key gaps remain in evaluating long-term stability under field conditions, operational feasibility in resource-limited settings, and a comprehensive cost-benefit analysis in comparison with existing point-of-care diagnostic technologies. Future efforts should prioritize rigorous field validation, stability assessment, scalability evaluation of production processes, and the development of a practical kit format to determine the true translational value of this method in clonorchiasis control programs.

## Supporting information

S1 Fig Expression and verification of CSTR1 antigen. (A) Expression and purification of CSTR1 antigen. 1: Whole bacteria of pET41a empty vector, 2: Supernatant of pET41a empty vector, 3: Precipitation of pET41a empty vector, 4: Whole bacteria of pET41a-CSTR1 plasmid, 5: Supernatant of pET41a-CSTR1 vector, 6: Precipitation of pET41a-CSTR1 vector (B) Purified antigens were identified using GST monoclonal antibodies.7: Supernatant of pET41a empty vector, 8: Purified CSTR1 (C) Western Blot analysis validated the reactivity of CSTR1 across multiple hosts. 9: Positive serum of rabbit infected with *C. sinensis*, 10: Negative serum of rabbit, 11: Positive serum of dog infected with *C. sinensis*, 12: Negative serum of dog, 13: Positive serum of human infected with *C. sinensis*, 14: Negative serum of human.
(TIF)

S2 Fig Preparation and verification of the three functional nanoflowers. (A) Microscopic morphology and detection effect of the HRP@Cu-HNFs. (B) Microscopic morphology and detection effect of the McAb@Cu-HNFs. (C) Microscopic morphology and detection effect of the McAb-HRP@Cu-HNFs.
(TIF)

S3 Fig Catalytic activity analysis of the nanoflowers. (A, B, C, D) Multi-host (rabbit, mouse, dog and human) serum detection via McAb-HRP@Cu-HNFs. (E) Storage stability testing of McAb-HRP@Cu-HNFs (F) The optimum operating PH of McAb-HRP@Cu-HNFs.
(TIF)

S1 Table Screening of antigen coating concentration and antibody dilution ratio by chessboard method. (DOCX)

S2 Table Comparison of dog and human sera samples by KK microscopy and nano-cELISA. (DOCX)

S3 Table The efficacy of serum ELISA techniques in detecting human clonorchiasis. (DOCX)
