# Supplementary figures and images for: One-pot nanoflower-based sensitive colorimetric biosensor for multihost detection of zoonotic clonorchiasis
Source: PLoS Negl Trop Dis. 2026 Apr 13;20(4):e0014197. doi: 10.1371/journal.pntd.0014197 (PMC13089884; doi:10.1371/journal.pntd.0014197)

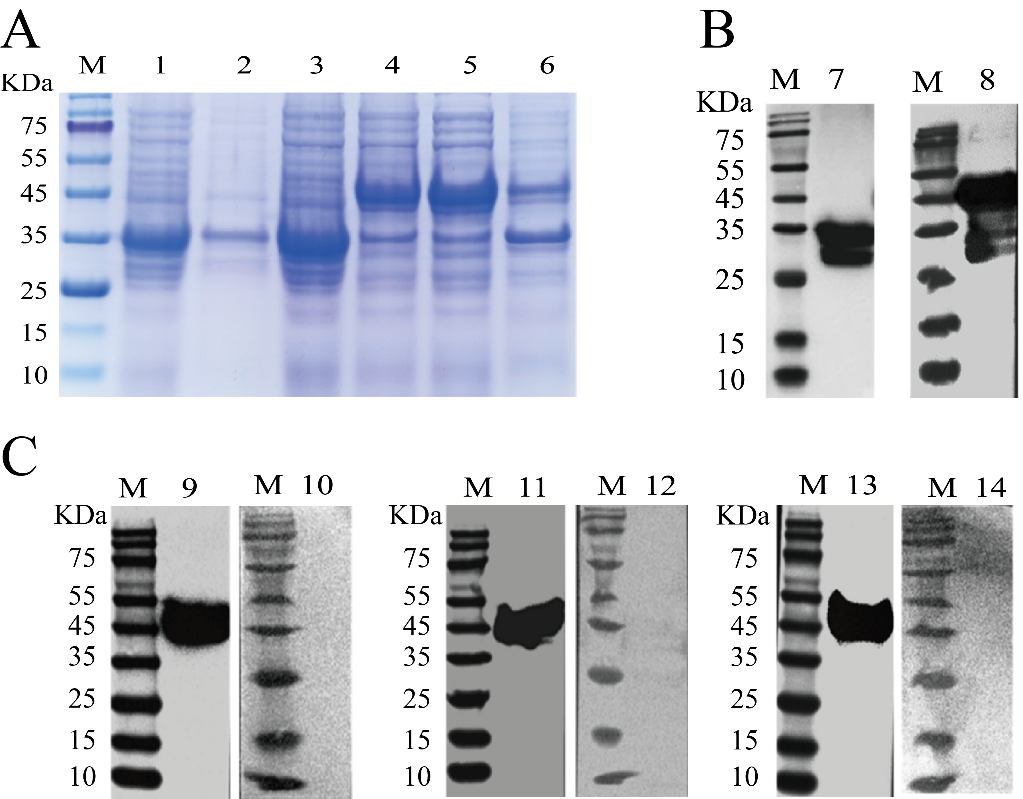

Supplement: S1 Fig — (A) Expression and purification of CSTR1 antigen. 1: Whole bacteria of pET41a empty vector, 2: Supernatant of pET41a empty vector, 3: Precipitation of pET41a empty vector, 4: Whole bacteria of pET41a-CSTR1 plasmid, 5: Supernatant of pET41a-CSTR1 vector, 6: Precipitation of pET41a-CSTR1 vector (B) Purified antigens were identified using GST monoclonal antibodies.7: Supernatant of pET41a empty vector, 8: Purified CSTR1 (C) Western Blot analysis validated the reactivity of CSTR1 across multiple hosts. 9: Positive serum of rabbit infected with C. sinensis, 10: Negative serum of rabbit, 11: Positive serum of dog infected with C. sinensis, 12: Negative serum of dog, 13: Positive serum of human infected with C. sinensis, 14: Negative serum of human. (TIF) [file pntd.0014197.s001.tif]

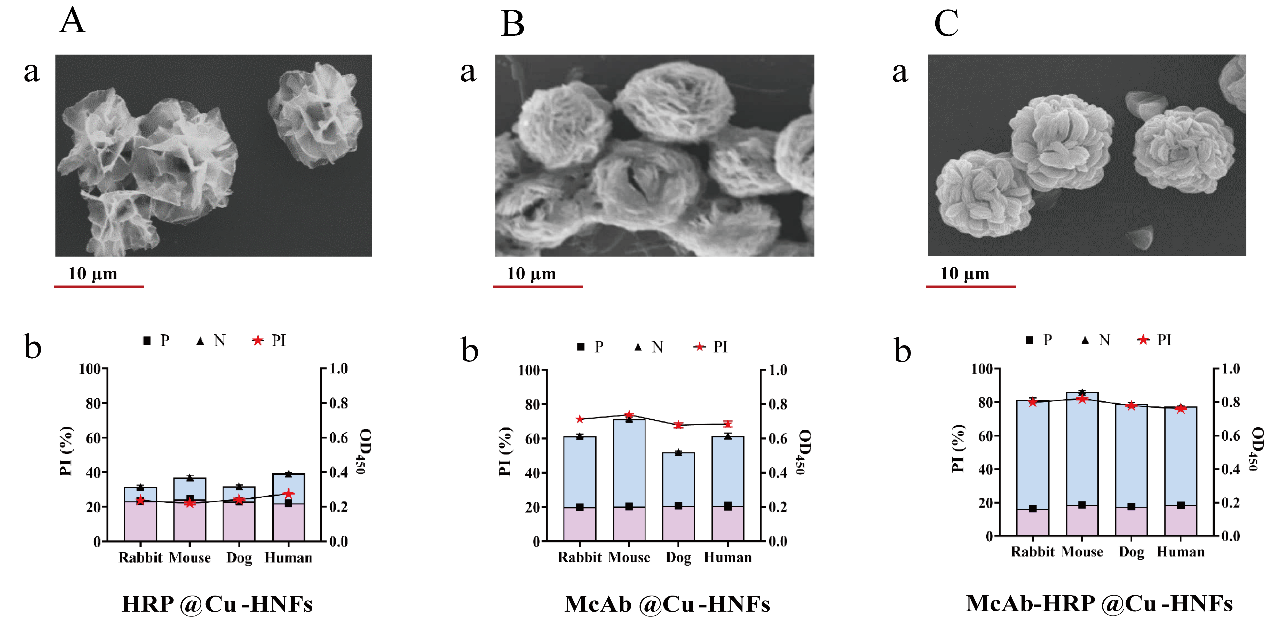

Supplement: S2 Fig — (A) Microscopic morphology and detection effect of the HRP@Cu-HNFs. (B) Microscopic morphology and detection effect of the McAb@Cu-HNFs. (C) Microscopic morphology and detection effect of the McAb-HRP@Cu-HNFs. (TIF) [file pntd.0014197.s002.tif]

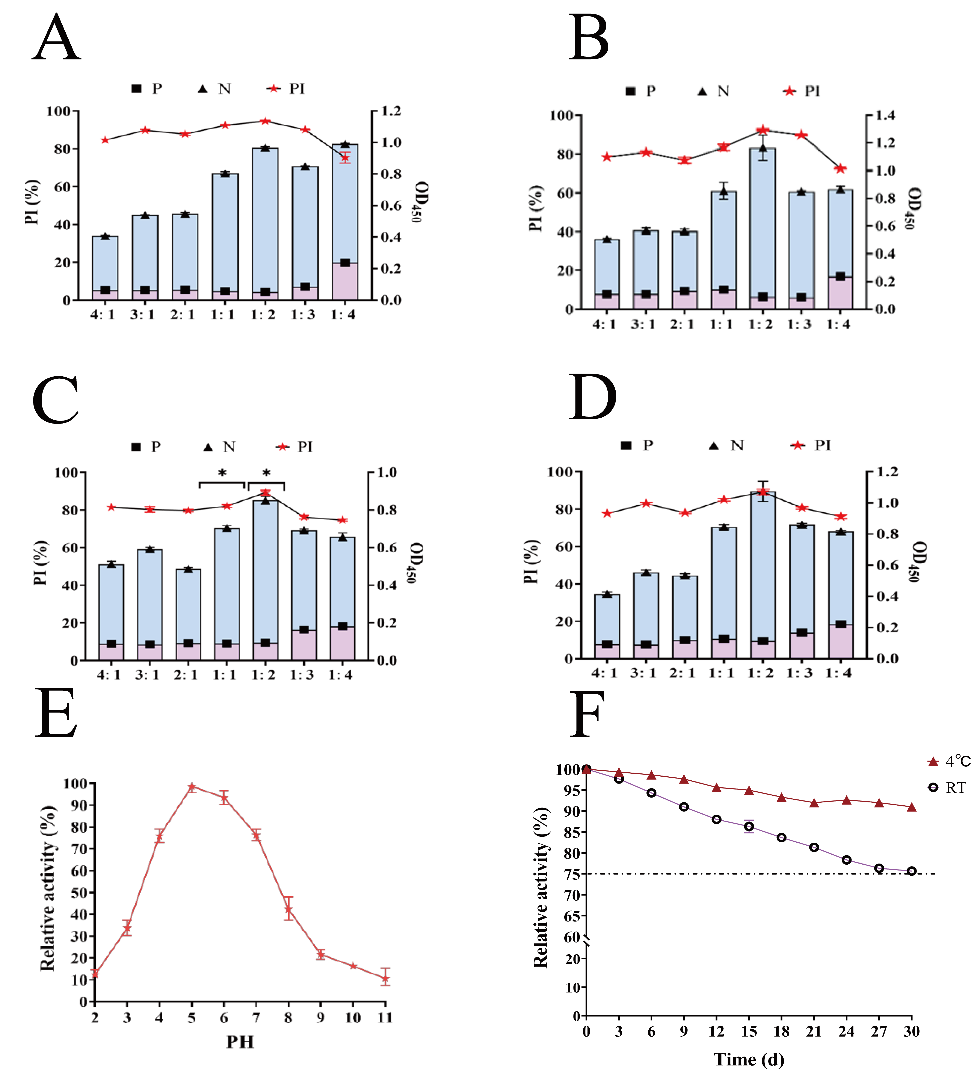

Supplement: S3 Fig — (A, B, C, D) Multi-host (rabbit, mouse, dog and human) serum detection via McAb-HRP@Cu-HNFs. (E) Storage stability testing of McAb-HRP@Cu-HNFs (F) The optimum operating PH of McAb-HRP@Cu-HNFs. (TIF) [file pntd.0014197.s003.tif]
