# Supplementary material for: One-pot nanoflower-based sensitive colorimetric biosensor for multihost detection of zoonotic clonorchiasis
Source: PLoS Negl Trop Dis. 2026 Apr 13;20(4):e0014197. doi: 10.1371/journal.pntd.0014197 (PMC13089884; doi:10.1371/journal.pntd.0014197)
Supplement: S1 Table — (DOCX) [file pntd.0014197.s004.docx]

**Table S1 Screening of antigen coating concentration and antibody dilution ratio by chessboard method.**

| Antigen coating（ug/mL） | PI radio | | | | |
| --- | --- | --- | --- | --- | --- |
|  | Sera: McAb-HRP@Cu-HNFs | | | | |
|  | 3：1 | 2：1 | 1：1 | 1：2 | 1：3 |
| 1.25 | 70.8% | 76.8% | 82.5% | 85.4% | 82.4% |
| 2.5 | 71.7% | 77.0% | 81.1% | 84.6% | 80.0% |
| 5 | 71.6% | 77.6% | 80.2% | 83.4% | 79.2% |
| **7.5** | 71.8% | 78.7% | 81.9% | **86.4**% | 81.0% |
| 10 | 74.2% | 78.5% | 81.9% | 85.1% | 80.9% |
