# Supplementary material for: One-pot nanoflower-based sensitive colorimetric biosensor for multihost detection of zoonotic clonorchiasis
Source: PLoS Negl Trop Dis. 2026 Apr 13;20(4):e0014197. doi: 10.1371/journal.pntd.0014197 (PMC13089884; doi:10.1371/journal.pntd.0014197)
Supplement: S2 Table — (DOCX) [file pntd.0014197.s005.docx]

**Table S2 Comparison of dog and human sera samples by KK microscopy and nano-cELISA**

| KK method  (Gold standard) | | Nano-cELISA (This study) | | | | | |
| --- | --- | --- | --- | --- | --- | --- | --- |
|  |  | Dogs | | Total | Humans | | Total |
|  |  | c_+_ | d- |  | e_+_ | f_-_ |  |
| a_+_ | 11 | | 0 | 11 | 59 | 4 | 63 |
| b_-_ | 1 | | 41 | 42 | 2 | 68 | 70 |
| Total | 12 | | 41 | 53 | 61 | 72 | 133 |
| g | 100%  95%CI [0.914-1.000] | | | | 94.44%  95%CI [0.864-0.985] | | |
| h | 91.67%,  95%CI [0.837-1.052] | | | | 96.72%  95%CI [0.887-0.996] | | |
| i | 98.11% | | | | 95.49% | | |

^a+^ KK method positive samples. ^b-^ KK method negative samples.

^c+^ Dog nano-cELISA positive samples. ^d-^ Dog nano-cELISA negative samples.

^e+^ Humans nano-cELISA positive samples. ^f-^ Humans nano-cELISA negative samples.

^g^ The calculated sensitivity of the nano-cELISA method.

^h^ The calculated specificity of the nano-cELISA method.

^i^ The calculated coincidence rate of the nano-cELISA method.
