# Supplementary material for: One-pot nanoflower-based sensitive colorimetric biosensor for multihost detection of zoonotic clonorchiasis
Source: PLoS Negl Trop Dis. 2026 Apr 13;20(4):e0014197. doi: 10.1371/journal.pntd.0014197 (PMC13089884; doi:10.1371/journal.pntd.0014197)
Supplement: S3 Table — (DOCX) [file pntd.0014197.s006.docx]

**Table S3 The efficacy of serum ELISA techniques in detecting human clonorchiasis**

| **Reference** | **Antigens** | **Sensitivity** | **Specificity** |
| --- | --- | --- | --- |
| 12 | Excretory-secretory antigen | 92.5% | 93.1% |
|  | Crude antigen | 88.2% | 87.8% |
| 45 | Recombinant CP | 93.3% | 93.3% |
| 47 | Recombinant CsCatL | 91.7% | 88.5% |
| 48 | Recombinant Cs 26GST | 50% | 93.2% |
| 49 | Recombinant CsTP 20.8 | 68% | 84% |
| 50 | Recombinant CsTP 21.1 | 95% | 98.8% |
| This  study | CsTR1 | 96.61%  [95%CI,  0.864-0.985] | 94.59%  [95% CI,  0.887-0.996] |
